# Reactivity of electrophilic cyclopropanes

**DOI:** 10.1515/pac-2023-0209

**Published:** 2023-04-25

**Authors:** Andreas Eitzinger, Armin R. Ofial

**Affiliations:** Department Chemie, Ludwig-Maximilians-Universität München, Butenandtstr. 5–13, 81377 München, Germany

**Keywords:** ICPOC-25, kinetics, linear free energy relationships, nucleophiles, ring-opening reactions, structure-reactivity

## Abstract

Cyclopropanes that carry an electron-accepting group react as electrophiles in polar, ring-opening reactions. Analogous reactions at cyclopropanes with additional C2 substituents allow one to access difunctionalized products. Consequently, functionalized cyclopropanes are frequently used building blocks in organic synthesis. The polarization of the C1–C2 bond in 1-acceptor-2-donor-substituted cyclopropanes not only favorably enhances reactivity toward nucleophiles but also directs the nucleophilic attack toward the already substituted C2 position. Monitoring the kinetics of non-catalytic ring-opening reactions with a series of thiophenolates and other strong nucleophiles, such as azide ions, in DMSO provided the inherent S_N_2 reactivity of electrophilic cyclopropanes. The experimentally determined second-order rate constants *k*_2_ for cyclopropane ring-opening reactions were then compared to those of related Michael additions. Interestingly, cyclopropanes with aryl substituents at the C2 position reacted faster than their unsubstituted analogues. Variation of the electronic properties of the aryl groups at C2 gave rise to parabolic Hammett relationships.

## Introduction

Additions of nucleophiles to the electron-deficient π-systems of Michael acceptors are among the best understood organic reactions and frequently used in organic synthesis for C–C- or C–X bond formations. Owing to their importance, the kinetics of Michael additions have extensively been investigated to characterize the electrophilic reactivities of electron-deficient π-systems [1], [2], [3], [4], [5].

**Fig. 1:**
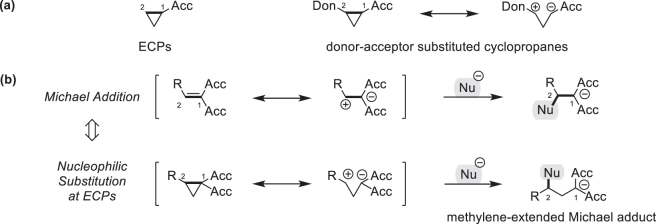
Reactivity of electrophilic cyclopropanes.

Also substituted cyclopropanes constitute a versatile class of compounds [6]. The reactivities of cyclopropanes are governed by the ring strain, which boosts the thermodynamic driving force of ring-opening reactions by more than 100 kJ mol^−1^ [7], as well as by the electronic nature of the substituents. Cyclopropanes with electron-accepting groups can thus be potent σ-electrophiles that undergo polar reactions with nucleophiles to give methylene-extended Michael adducts [8], [9], [10]. To further enhance the synthetic versatility of electrophilic cyclopropanes (ECPs) electron-donating groups can be installed at C2 of the cyclopropane ring (Fig. 1a). The manifold of options for chemical transformations of such donor-acceptor substituted cyclopropanes has been reviewed frequently [11], [12], [13], [14], [15], [16], [17], [18], [19], [20], [21] and includes various modes for the preparation of carbo- [22, 23] and heterocycles [24, 25]. Additionally, (3 + 2), (3 + 3), and (4 + 3) cycloadditions [26], [27], [28] as well as rearrangements [29, 30] and ring-opening reactions [18] of functionalized cyclopropanes offer access to many, structurally diverse building blocks.

Despite their key role in organic synthesis, a reliable and broadly applicable characterization of the polar reactivity of ECPs had not been available until recently. Previous kinetic studies by McKinney et al. and Ohkata et al. focused on a few reactions of dimedone- and Meldrum’s acid derived ECPs with pyridines [31, 32]. More recently, the Werz group used *in situ* ^19^F NMR kinetics to survey substituent effects on the reactivity of SnCl_4_-complexed 1,1-di(alkoxycarbonyl)-substituted cyclopropanes in (3 + 2)-cycloadditions with *p*-fluorobenzaldehyde [33, 34], which had been reported to follow a complex mechanism [35, 36].

We were intrigued, however, by simple ring-opening reactions of nucleophiles with ECPs because of their relation to Michael additions [9] and nucleophilic substitutions at sp^3^-hybridized carbon centers (Fig. 1b). It can be anticipated that kinetic studies of uncatalyzed ECP-nucleophile reactions under standardized conditions will provide fundamental insights into the factors that control polar cyclopropane reactivity and would be a valuable contribution to the understanding of σ-electrophilicity.

For thousands of electrophile-nucleophile reactions H. Mayr and co-workers have demonstrated that experimentally determined reactivities for polar, uncatalyzed reactions can, in general, be analyzed by using the Mayr–Patz eq. 1 [37], [38], [39], [40], [41], [42], [43].

(1) $$\text{Mayr}-\text{Patz}\;\text{equation}:\;\mathrm{lg}k_{2}\left(20\;{}^{\circ}C\right)=s_{\text{N}}\left(N+E\right)$$

In eq. 1, the electrophile’s strength is characterized by an electrophilicity descriptor *E*, and the reactivity of a nucleophile in a certain solvent is expressed by the nucleophilicity (*N*) and a susceptibility factor (*s*_N_). Equation 1 relies on a backbone of benzhydrylium ions and structurally related *p*-quinone methides as reference electrophiles but is not limited to these classes of reactants [44]. At present, Mayr’s reactivity scales cover more than 1250 nucleophiles and over 350 electrophilic species [45]. Hence, these scales provide a unique collection of systematically calibrated reactivity data, which facilitate the prediction of feasible reactions on a semi-quantitative fundament. Equation 1 enables users to calculate second-order rate constants that are usually accurate within a factor of 10–100 if compared to experimentally determined values for *k*_2_ [40], [41], [42, 46]. Within a reactivity range that spans >40 orders of magnitude in organic synthesis this degree of reliability is sufficient, and eq. 1 has become an established and useful tool for the design of novel organic synthesis [47], [48], [49], [50], [51].

In this context, we set out to investigate the inherent electrophilic reactivity of ECPs through kinetic studies of their reactions with a series of highly nucleophilic thiophenolates. Ring-opening S_N_2 reactions, in which bond formation and bond breaking are coupled events cannot be expected to follow the simple three-parameter eq. 1 that was designed for electrophile-nucleophile reactions, in which only one new σ-bond is formed. Still, previous examples showed that relative nucleophilicities, as expressed by the Mayr *N* parameter, often also hold for reactions with electrophilic S_N_2 substrates [52, 53], in particular when the type of atom at the nucleophilic center is kept constant.

## Nucleophilicities of thiophenolates

Sufficiently reactive nucleophiles with UV–vis absorption are needed to study the kinetics of their reactions with cyclopropanes at a timescale that is appropriate for efficient photometric measurements. Beneficially, such nucleophiles could be fine-tuned in reactivity by electronic effects without changing the steric demand at the nucleophilic reaction center.

**Fig. 2:**
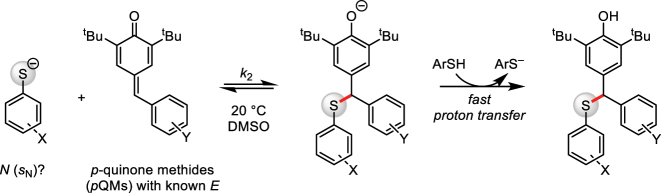
Reactions of X-substituted thiophenolates with *p*QMs (reference electrophiles).

Interestingly, sodium thiophenolate had been reported to react with the Meldrum’s acid derived spirocyclopropane already at room temperature [8]. We, therefore, set out to determine the Mayr nucleophilicity parameters *N* and *s*_N_ of a set of thiophenolates by following their reactions with *p*-quinone methides (*p*QMs, Fig. 2) [54]. The *p*QMs are neutral, structural analogs of the positively charged benzhydrylium ions that serve as reference electrophiles for the characterization of neutral nucleophiles [44, 55]. Thus, *p*QMs logically extend the series of reference electrophiles used to construct Mayr’s nucleophilicity scales and allow one to reliably calibrate the reactivity of anionic nucleophiles [56], [57], [58], [59], [60].

Adduct formation of *p*QMs with thiophenolates is reversible in DMSO, however, which gave rise to low conversions under the conditions of the kinetic measurements, which were performed at low reactant concentrations (5 × 10^−5^ to 2 × 10^−3^ mol dm^−3^) [54]. To generate the neutral phenol products in fast, thermodynamically favorable reactions from the intermediate phenolates, an excess of thiophenols was applied in the kinetic measurements. Owing to the higher Brønsted acidity of thiophenols (p*K*_a_ < 11.2 [61]) compared to 2,6-di-tert-butylphenol (p*K*_a_ = 17.3 in DMSO [62]), the thiophenols efficiently trapped the intermediate phenolate-type adducts by fast protonation and regenerated the consumed amount of the nucleophilic thiophenolate.

**Fig. 3:**
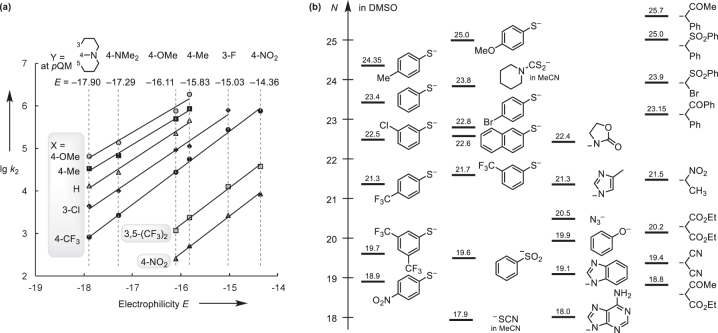
Reactivities of thiophenolates in DMSO. (a) Determination of the nucleophile-specific reactivity parameters *N* and *s*_N_ for X-substituted thiophenolates from the linear relationships of lg *k*_2_ with the electrophilicities *E* of the *p*QMs (with data from [54]). (b) Section of the Mayr nucleophilicity (*N*) scale with entries for S-, O-, N- and C-centered nucleophiles (in DMSO).

Under these conditions, second-order rate constants, *k*_2_ (dm^3^ mol^−1^ s^−1^), could be determined by the photometric monitoring of the reactions of sodium or potassium thiophenolates with the *p*QMs in DMSO at 20 °C. In a next step, nucleophile-specific reactivity parameters *N* (and *s*_N_) for the thiophenolates were calculated by applying the rate constants *k*_2_ and the known electrophilicity parameters *E* of the *p*QMs in the Mayr–Patz eq. 1 (Fig. 3) [54]. As shown in Fig. 3a, lg *k*_2_ of reactions of thiophenolates with *p*QMs correlated linearly with the respective electrophilicities *E*, which enabled us to determine the nucleophilicities *N* of the thiophenolates from the intercepts on the abscissa. The slopes (*s*_N_) of the nucleophile-specific correlation lines reflect the susceptibility of the nucleophiles towards changes in the electrophilic reaction partner.

Thiophenolates cover a range of 6 logarithmic units in Mayr’s nucleophilicity scale (Fig. 3b). The reactivities of the 4-methyl- and 4-methoxy-substituted thiophenolates exceed even those of piperidine-1-carbodithioate (*N* = 23.8 in MeCN [63]), which previously set the benchmark for the most reactive S-nucleophile in the *N* scale. The depicted section of the Mayr nucleophilicity scale in Fig. 3b, furthermore, permits a semi-quantitative comparison of the reactivities of thiophenolates with those of structurally diverse O-, N- and C-centered nucleophiles, which are located at a similarly high reactivity level [45].

**Fig. 4:**
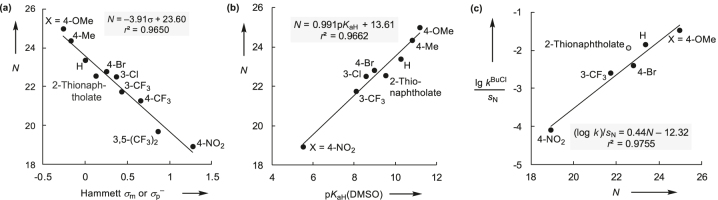
Linear correlations of the nucleophilicities *N* of thiophenolates with Hammett substituent parameters *σ*_m_ and *σ*_p_^–^ (a) and Brønsted basicities p*K*_aH_(DMSO) (b) as well as of S_N_2 reactivities (vs. BuCl) with nucleophilicities *N* of thiophenolates (c) [the entry for 2-thionaphtholate (open circle) is shown for comparison but was not used to construct the correlation line in Fig. 4c].

The thiophenolate nucleophilicities *N* are mainly controlled by the electronic properties of the ring substituents in meta- or para-positions, in accord with the excellent linear correlation of *N*(ArS^−^) with the Hammett substituent constants (*σ*_m_ and *σ*_p_^–^ [64]) in Fig. 4a. It has been shown several times before, that reactivities of structurally diverse nucleophiles do not necessarily correlate well with Brønsted basicity [65, 66]. However, owing to the systematic variation of the electron density at the reaction center, the linear relationship in Fig. 4b illustrates that the nucleophilicities *N* of the ring-substituted thiophenolates, including 2-thionaphtholate, systematically increase with increasing Brønsted basicity, which is expressed by the acidities of the corresponding thiophenols p*K*_aH_(DMSO) [54].

Most interestingly, the nucleophilicities *N* of the thiophenolates reflect not only their reactivity towards Michael acceptors, such as *p*QMs, for which they were calibrated [54], but also towards S_N_2 substrates. For example, Bordwell and Hughes had determined the rate constants for reactions of thiophenolates with 1-chlorobutane (BuCl) in DMSO at 25 °C [61]. Fig. 4c demonstrates that the reactivity of thiophenolates towards BuCl, which is a prototypical example for an electrophile in S_N_2 reactions, is well described by their nucleophilicity parameters *N* and *s*_N_. Owing to the expedient UV–vis absorption of the thiophenolates [54], we investigated their capacity as reference nucleophiles in kinetic studies to characterize colorless electrophiles, such as sufficiently activated ECPs.

## Reactions of thiophenolates with electrophilic cyclopropanes

### Product studies

**Fig. 5:**
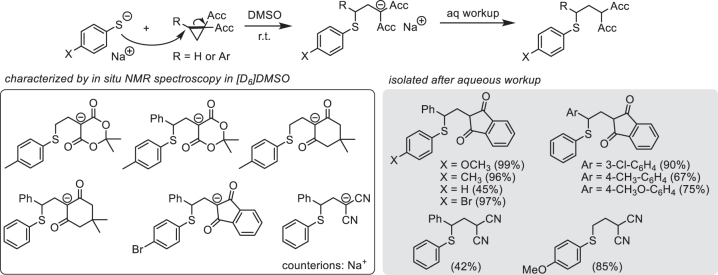
Ring-opening reactions of electrophilic cyclopropanes with thiophenolates (data from [67, 68]) (Experimental procedure and analytical data for 2-(2-((4-methoxyphenyl)thio)ethyl)malononitrile [68]: A mixture of *p*-thiocresol (70.1 mg, 0.500 mmol) and potassium *tert*-butoxide (58.9 mg, 0.525 mmol) dissolved in DMSO (2 mL) was added to a solution of cyclopropane-1,1-dicarbonitrile (46.1 mg, 0.501 mmol) in DMSO (3 mL). The reaction mixture was stirred for 30 min at ambient temperature (ca. 22 °C). Then, an aqueous ammonium chloride solution (10 mL) was added. The mixture was extracted with diethyl ether (3 × 20 mL). The combined organic layers were washed with brine (20 mL), dried over magnesium sulfate, and the volatiles were removed in the vacuum. Purification by column chromatography (silica gel, eluent: pentane/ethyl acetate = 3/1, v/v) furnished 98.8 mg of 2-(2-((4-methoxyphenyl)thio)ethyl)malononitrile (yield: 85 %) as a viscous oil. ^1^H NMR (599 MHz, CDCl_3_): δ 7.37 (d, *J* = 8.8 Hz, 2 H), 6.88 (d, *J* = 8.8 Hz, 2 H), 4.15 (t, *J* = 7.4 Hz, 1 H), 3.81 (s, 3 H), 3.02 (t, *J* = 6.7 Hz, 2 H), 2.22 (q, *J* = 6.9 Hz, 2 H). ^13^C NMR (151 MHz, CDCl_3_): δ 160.1, 134.6, 123.0, 115.3, 112.3, 55.5, 32.6, 30.4, 20.8.).

First, we investigated the products of ECP-thiophenolate reactions (Fig. 5). Mixing sodium thiophenolates with spiro-activated or 1,1-dicyano-substituted cyclopropanes in a 1:1 ratio in [D_6_]DMSO produced stable solutions of sodium enolates (or dicyano-stabilized carbanions) by cyclopropane-ring opening reactions. Aqueous workup of the ionic adducts gave methylene-extended Michael adducts [67]. NMR characterization of the ionic adducts as well as of isolated products showed that thiophenolates had reacted with 2-arylated cyclopropanes regioselectively at the already substituted C2 position of the three-membered ring [67]. The excellent chemo- and regioselectivity of the investigated electrophile-nucleophile combinations showed that photometrical monitoring of the uncatalyzed reactions of ECPs with thiophenolates would allow us to get insight in the kinetics of the ring-opening reaction of spiro-activated or 1,1-dicyano-substituted cyclopropanes. ECPs with 1,1-bis(ethoxycarbonyl) substituents had to be excluded from further kinetic studies because they did not undergo clean ring-opening reactions with thiophenolates but furnished complex mixtures of products presumably resulting from nucleophilic attack at the carbonyl C-atoms of the ester groups as well as from anionic polymerization.

### Kinetic studies

To assess the inherent electrophilic reactivities of cyclopropanes towards nucleophilic attack, the kinetics of ring-opening reactions of ECPs with thiophenolates in DMSO at 20 °C were monitored by using stopped-flow or conventional UV–vis spectrophotometry. The DMSO solutions of the sodium and potassium thiophenolates employed herein have absorption maxima *λ*_max_ = 302–329 nm [54]. Under pseudo-first-order conditions, that is, with the ECP in at least ten-fold excess, monoexponential decays of the thiophenolate absorbances were detected. The (pseudo-)first-order rate constants *k*_obs_ (s^−1^) were determined by fitting the exponential function *A* = *A*_0_ exp(–*k*_obs_ *t*) + *C* to the recorded absorption vs. time curve. Second-order rate constants *k*_2_ (dm^3^ mol^−1^ s^−1^) were obtained as the slopes of the linear correlations of *k*_obs_ (s^−1^) vs. thiophenolate concentration (mol dm^−3^) [67, 68].

For 1,3-indandione-derived ECPs, the increase of the absorption at 390–396 nm was monitored because the reactions of the thiophenolates with this type of electrophile produced stable solutions of UV-light absorbing ionic adducts. As before, the ECPs were used as the excess compounds and *k*_2_ was derived from the slope of the linear relationship of *k*_obs_ vs. ECP concentration [67].

**Fig. 6:**
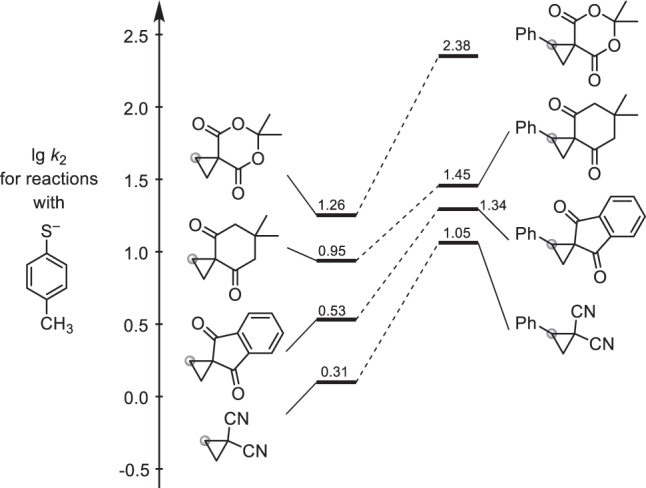
Reactivities of ECPs towards the common reference nucleophile *p*-thiocresolate (DMSO, 20 °C, data from [67, 68], see also Table 1, the site of nucleophilic attack at the ECPs is highlighted by a grey dot).

As a result, the reactivities of ECPs could be benchmarked by comparing the lg *k*_2_ values for their reactions with a common reference nucleophile. In Fig. 6, the *p*-thiocresolate ion serves as the common nucleophilic reaction partner for comparing the structural effects on the reactivity of spiro- or 1,1-dicyano-activated ECPs with and without phenyl substituent at the C2 position of the cyclopropane ring.

To note, ECPs carrying only one acceptor group did not take part in uncatalyzed ring-opening reactions with thiophenolates at 20 °C, underscoring the need for activating catalysts to achieve synthetically useful reactions with these less reactive ECPs. As a consequence, 1,1-bis(acceptor)-substituted ECPs are shown in Fig. 6. In particular, ECPs with spirocyclic acceptor groups reacted rapidly with thiophenolates. In spiro-activated ECPs, the (alkoxy)carbonyl groups are locked orthogonally with respect to the cyclopropane plane which facilitates charge delocalization in the transition state [8, 9, 67]. Also ECPs with 1,1-dicyano substitution reacted efficiently as a result of the cyclindrical electron-accepting π-systems, which are always in the right orientation to stabilize negative charge at the neighbor carbon atom [67, 68].

As shown in Fig. 6, Meldrum’s acid-derived cyclopropanes generally reacted faster than their dimedone analogues. The enhanced electrophilicities of those cyclopropanes, which were attached to Meldrum’s acid, are rationalized by the fixed s-(*E*) ester conformation in the ‘double lactone’ moiety. In this conformation, favorable *n*_O_ → σ*(CO) interactions, which typically stabilize open-chain esters with free rotation around the C–O single bond, are ineffective [69]. Accordingly, analogous reactivity trends with enhanced electrophilic reactivity of lactone derivatives over structurally comparable open-chain esters have also been reported for hydrolysis reactions of lactones and esters themselves [70] as well as for nucleophilic additions to α,β-unsaturated ester and lactone derivatives [5, 71].

Independent of the electron-accepting groups at C1, all types of ECPs in Fig. 6 are strongly activated by a phenyl group in C2 position of the cyclopropane unit, which is reminiscent of the benzyl effect in S_N_2-type reactions [72], [73], [74], [75] and in accord with observations by McKinney and co-workers who studied ring-opening reactions of cyclopropanes by pyridine [31]. The ECPs with a phenyl group reacted up to 15 times faster than those without the C2 substituent [67]. The true activating effect of the 2-Ph groups must even be greater because the observed reactivities only reflect a part of the activation which is counterbalanced by the increase in steric hindrance at the reaction center.

**Fig. 7:**
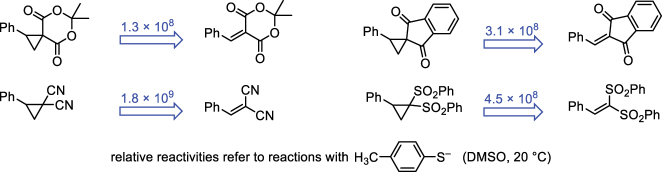
Comparing the ECP reactivities (Table 1) with those of structurally related Michael acceptors [rate constants for Michael acceptors were calculated by applying *N* (24.35) and *s*_N_ (0.69) for the *p*-thiocresolate ion and electrophilicities *E* from ref [4] in the Mayr–Patz eq. 1].

With identical acceptor groups attached to either a cyclopropane or the π-system of a Michael acceptor, the Michael acceptors are by 8–9 orders of magnitude more reactive towards nucleophiles than their structurally analogous counterpart in the ECP series (Fig. 7) [67, 68]. As discussed above, ECPs are activated by phenyl groups at the electrophilic center (cf. Fig. 6). The contrary has been observed for Michael acceptors, however, which have been shown to drop in electrophilic reactivity by 4–5 units in Mayr’s electrophilicity scale when the terminal = CH_2_ group of the π-system is changed to a = CHPh group [4].

### Correlation analysis for thiophenolate-ECP reactions

**Fig. 8:**
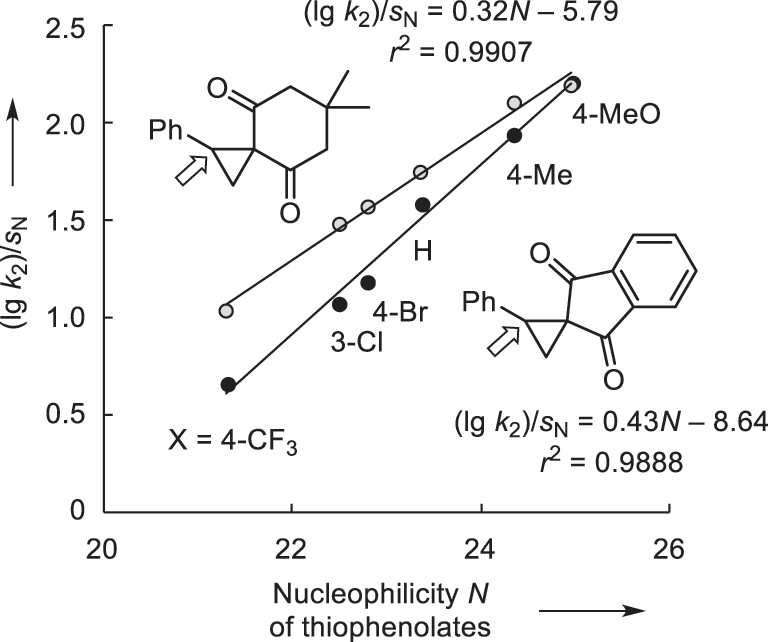
Mayr plot of reactivity data for reactions of X-substituted thiophenolates with 2-phenyl substituted spiro-activated ECPs (the site of nucleophilic attack at the ECPs is marked by an arrow, with data from [67]).

The Mayr–Patz eq. 1 cannot be used to predict nucleophile-ECP kinetics in general because it was calibrated for simple addition reactions in which only one new σ-bond is formed between the electrophile and the nucleophile. Given that they follow an S_N_2 mechanism, ring-opening reactions at ECPs fulfill the condition to involve formation of one new σ-bond. However, this process is inseparably coupled with breaking the C1–C2 σ-bond in the cyclopropane. Still, linear correlations of excellent quality were obtained when (lg *k*_2_)/*s*_N_ were correlated with the thiophenolates’ *N* parameters (Fig. 8) [67]. The slopes of the correlation lines deviate from unity, however, in accord with previous findings for S_N_2 reactions [52]. Thus, classical Mayr *E* parameters for ECPs cannot be calculated which hampers the straightforward comparison of ECP reactivities with those of other classes of electrophiles.

Second-order rate constants for the reactions of phenolates (ArO^−^) with electrophiles showed little dependence on the nature of polar, aprotic solvents, and rate constants for reactions in DMF, acetonitrile, and DMSO were within one order of magnitude [76]. On this basis, we can assume that also the reactivities of thiophenolates (ArS^−^) that we determined in DMSO solution will not vary significantly if other polar, aprotic solvents are used. Therefore, correlations, as depicted in Fig. 8, will at least enable users to calculate reaction times for ECP reactions with further thiophenolates in polar, aprotic solvents, such as DMSO, DMF, or acetonitrile.

The accelerating effect of adjacent π-systems on rates of S_N_2 reactions has been investigated thoroughly on several occasions and is a result of both (hyper)conjugative stabilization and favorable electrostatic interactions between the reaction partners, which are predominant when negatively charged nucleophiles are involved [72], [73], [74], [75]. The same arguments apply to ring-opening reactions of ECPs by thiophenolates (cf. Fig. 6).

**Fig. 9:**
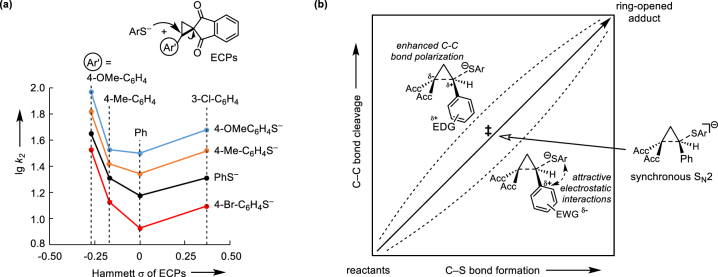
Analyzing the kinetics of ECP ring-opening reactions. (a) Parabolic Hammett plots for the kinetics of the reactions of thiophenolates with 1,3-indandione-activated cyclopropanes which carry electron-rich and electron-poor aryl groups Ar’ at the C2 position (with data from [67]). (b) Schematic depiction of TS stabilizing interactions in S_N_2 reactions of 2-aryl substituted ECPs with thiophenolates.

Systematic variation of substituents at the 2-phenyl group of indandione-derived cyclopropanes resulted in parabolic Hammett plots (Fig. 9a) with a minimum of reactivity for the 2-phenyl-substituted ECP. Electron-withdrawing as well as electron-donating substituents at the 2-aryl group enhanced the reactivity of the ECP [67], in analogy to a report by Hudson and Klopman who observed a parabolic Hammett correlation when analyzing the kinetics of S_N_2 reactions of thiophenolates with benzylbromides (in methanol, 20 °C) [77].

The More O’Ferrall-Jencks diagram [78, 79] in Fig. 9b visualizes that the transition state stabilization is influenced by the properties of the ECP. Electron-withdrawing groups (EWG) enhance the rate of the ring-opening reaction by favorable electrostatic interactions with anionic nucleophiles. However, also electron-donating groups (EDG) accelerate the reaction because they induce a stronger polarization of the C1–C2 bond. Furthermore, the polarized C1–C2 bond also guides regioselectivity of nucleophilic attack toward the already substituted C2 position.

### Comparison of ECP reactivities towards anionic S- and N-nucleophiles

In order to explore whether the reactivity trends observed in reactions of ECPs with thiophenolates also hold for combinations of ECPs with other types of nucleophiles, we determined the kinetics of ECP ring-opening reactions with the azide ion. First reported by Alvarez and co-workers for Meldrum’s acid-derived cyclopropanes [80], ring-opening reactions of cyclopropanes by azide ions have since also been studied by Kerr et al. [81]. In synthetic studies by Trushkov and coworkers, an excess of the azide salt, the presence of a proton source (Et_3_N · HCl), and elevated temperatures were required for good yields and acceptable reaction times to furnish ring-opened adducts of 1,1-bis(alkoxycarbonyl)-substituted cyclopropanes [82]. Most interestingly, the indanone-derived ECP **4** and the 1,1-dicyano-activated ECP **6** were reported to undergo ring-opening by sodium azide already at 25 °C (for **4**: 85 % yield after 6 h reaction time; for **6**: 43 % yield from a 1:1:1 mixture of **6**/NaN_3_/Et_3_N·HCl after 3 h reaction time) [82]. Furthermore, nucleophile-specific Mayr reactivity parameters of *N* = 20.50 and *s*_N_ = 0.59 were reported for the azide ion in DMSO solution [83], which allowed us to hypothesize that following the kinetics of ECP ring-opening reactions with azide ions would be experimentally accessible under our standard conditions, that is, at 20 °C in DMSO.

**Fig. 10:**
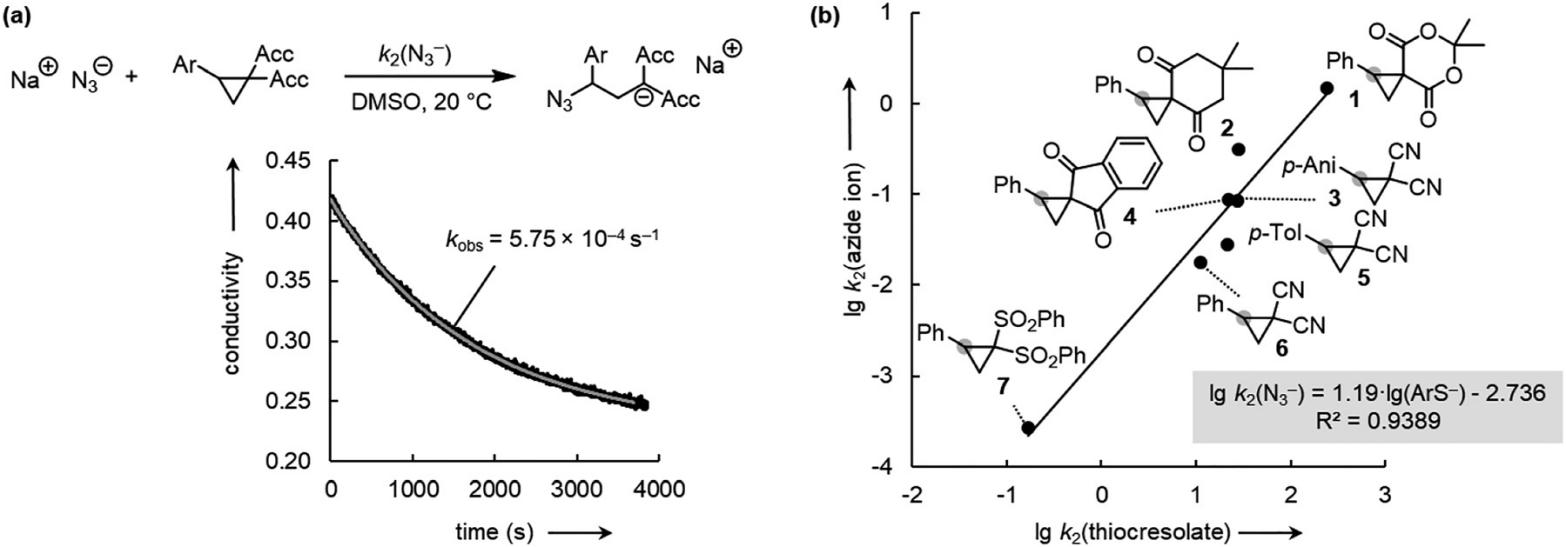
Kinetics of the reactions of ECPs with azide ions. (a) Exponential decrease of conductivity in the course of the azide addition reaction to ECPs (exemplified for the reaction of NaN_3_ with ECP **6** (15 equivs.), [N_3_^−^]_0_ = 1.86 × 10^−3^ mol dm^−3^, [**6**]_0_ = 2.79 × 10^−2^ mol dm^−3^) [68]. (b) Linear correlation of the reactivities (lg *k*_2_) of ECPs **1**–**7** towards the azide ion (N_3_^−^) and the *p*-thiocresolate ion (DMSO, 20 °C, the site of nucleophilic attack at the ECPs is highlighted by a grey dot) [68]; *p*-Ani = *p*-anisyl, *p*-Tol = *p*-tolyl.

We found that upon mixing 2-aryl substituted ECPs with an excess of sodium azide in [D_6_]DMSO at 20 °C, ionic adducts formed exclusively and in high yields within minutes to hours (Fig. 10a). Conductometry was a suitable method for following the kinetics of the ring-opening reactions because the initial sodium azide solutions had significantly higher molar conductivities in DMSO than the azide-ECP adduct solutions at the end of the reactions. Thus, the decay of conductivity after addition of an ECP (in excess) to a sodium azide solution could be monitored to obtain first-order rate constants *k*_obs_ (s^−1^) (Fig. 10a). The second-order rate constants *k*_2_ (N_3_^−^) were then derived as the slopes of the linear correlations in plots of *k*_obs_ vs. ECP concentration. As summarized in Table 1 and depicted in Fig. 10b, the relative reactivities of the investigated ECPs towards azide ions followed the same trend as observed with the S-nucleophile *p*-thiocresolate.

**Table 1:** Second-order rate constants *k*_2_ (dm^3^ mol^−1^ s^−1^) for the reactions of 2-aryl substituted ECPs **1**–**7** with *p*-thiocresolate (ArS^−^) and azide ions (N_3_^−^) in DMSO at 20 °C [67, 68]; structures of ECPs **1**–**7** are shown in Fig. 10b.

ECP	*k*_2_ (ArS^−^)	*k*_2_ (N_3_^−^)
**1**	2.42 × 10^2^	1.49
**2**	2.82 × 10^1^	3.08 × 10^−1^
**3**	2.74 × 10^1^	8.58 × 10^−2^
**4**	2.19 × 10^1^	8.82 × 10^−2^
**5**	2.16 × 10^1^	2.80 × 10^−2^
**6**	1.13 × 10^1^	1.76 × 10^−2^
**7**	1.67 × 10^−1a^	2.69 × 10^−4^

^a^Extrapolated from a Hammett plot based on rate constants for reactions with three less reactive thiophenolates.

## Conclusions and outlook

After characterization of the nucleophilicities of thiophenolate ions in DMSO by their *N* (and *s*_N_) parameters in the framework of Mayr’s reactivity scales [54], this type of highly reactive, anionic nucleophiles was used to investigate the reactivity of ECPs through ring-opening reactions. Photometrical monitoring of the kinetics of non-catalytic ring-opening reactions of thiophenolates in DMSO provided the inherent S_N_2 reactivity of electrophilic cyclopropanes [67].

As long as steric hindrance is influencing the adduct formation only moderately, the thiophenolate-derived reactivity ranking of ECPs can straightforwardly be used to predict relative reactivities of ECPs towards further types of anionic nucleophiles. This has exemplarily been shown for the kinetics of azide-ECP adduct formations, which were experimentally determined by conductometric methods [68].

The thiophenolate-ECP data allow one to compare the ECP reactivities with those of analogously substituted Michael acceptors, which are by factors of 10^8^–10^9^ stronger electrophiles. Different from reactivity trends in the series of Michael acceptors, which are strongly deactivated by β-phenyl groups at the olefinic π-system [4], ECPs react faster when they carry an aryl group at the C2 position of the cyclopropane [67]. Variable types of transition state stabilizing effects gave rise to parabolic Hammett plots for ECPs with differently substituted aryl groups at the C2 of the cyclopropane unit [67].

Currently, we are trying to expand the kinetic measurements with ECPs to further classes of anionic nucleophiles, including carbon-centered reactants. We hope that such experimental kinetic data will induce further synthetic applications of ECPs in uncatalyzed reactions. Furthermore, standardized experimental kinetic data have become increasingly important to serve as input for statistical reaction analysis [84, 85] or the training of self-learning algorithms, and we look forward to seeing improved quality of rate predictions generated by machine learning methods [86], [87], [88], [89], [90] or the application of neural networks [91].
